# Effects of Extreme Weather Events on Nitrous Oxide Emissions from Rice-Wheat Rotation Croplands

**DOI:** 10.3390/plants13010025

**Published:** 2023-12-20

**Authors:** Ye Xia, Congsheng Fu, Aimin Liao, Huawu Wu, Haohao Wu, Haixia Zhang

**Affiliations:** 1 Key Laboratory of Watershed Geographic Sciences, Nanjing Institute of Geography and Limnology, Chinese Academy of Sciences, Nanjing 210008, China; yxia@niglas.ac.cn (Y.X.);; 2 Collaborative Innovation Center on Forecast and Evaluation of Meteorological Disasters (CIC-FEMD), Nanjing University of Information Science & Technology, Nanjing 210044, China; 3 Chuzhou Scientific Hydrology Laboratory, Nanjing Hydraulic Research Institute, Chuzhou 239080, China

**Keywords:** rice-wheat cropland, extreme weather event, field observation, community land model

## Abstract

Cropland ecosystems are significant emission sources of N_2_O, but a limited number of studies have focused on the impact of extreme weather events on N_2_O fluxes from cropland. This present study integrated field observations and model simulations to explore the responses of N_2_O fluxes to extreme weather events in typical rice and wheat rotation croplands in the middle and lower reaches of the Yangtze River (MLRYR) in China. The findings revealed that the studied rice-wheat rotation cropland exhibited a net source of N_2_O over the three-year monitoring period, with annual cumulative N_2_O emissions ranging from 190.4 to 261.8 mg N m^−2^. N_2_O emissions during the rice and wheat growing seasons accounted for 29% and 71% of the total yearly emissions, respectively. Extreme heat events led to a 23% to 32% increase in observed N_2_O emissions from cropland. Observed N_2_O emissions from irrigated rice fields during extreme precipitation events were 45% lower than those during extreme drought events. In contrast, extreme precipitation events raised observed N_2_O emissions from rain-fed wheat fields by 36% compared to the multi-year average, while extreme drought events reduced N_2_O emissions from wheat fields by 20%. Regional simulations indicated that annual cumulative N_2_O emissions from croplands in the MLRYR are projected to increase from 207.8 mg N m^−2^ under current climate to 303.4 mg N m^−2^ in the future. Given the episodic nature and uncertainties associated with N_2_O emissions from cropland, further validation is necessary for utilizing the model to explore the effects of extreme weather events on N_2_O in cropland ecosystems.

## 1. Introduction

Nitrous oxide (N_2_O) is a potent greenhouse gas that not only exhibits a strong ozone-depleting potential, but also has the ability to persist in the troposphere for an extended period [1,2]. With a warming potential about 300 times greater than CO_2_, N_2_O concentration in the atmosphere was approximately 331 ppb in 2018, and it continues to rise at a rate of 4 ppb per five years [3,4,5]. Cropland ecosystems are recognized as significant contributors to N_2_O emissions in terrestrial ecosystems [6,7]. The annual N_2_O emissions from cropland ecosystems account for nearly 70% of the world’s total N_2_O emissions and 42% of anthropogenic emissions [8,9]. Hence, accurate monitoring and assessment of N_2_O fluxes in cropland ecosystems are of great importance in the context of global warming. 

With the ongoing climate change, extreme weather events such as storms, floods, heat waves, and droughts are becoming more frequent, and these events have been reported to destabilize cropland ecosystems, influence nutrient cycles, and affect seasonal dynamics of N_2_O fluxes in croplands [10,11,12]. Particularly, extreme weather events such as extreme precipitation, drought, and heat can modify soil water content, soil temperature, and evapotranspiration. These changes subsequently affect nitrification and denitrification processes, as well as microbial activity in the soil, ultimately impacting N_2_O fluxes [13]. Furthermore, extreme weather events indirectly affect N_2_O fluxes in cropland ecosystems by limiting crop growth through their impacts on photosynthesis and respiration in the field, thus reducing plant nitrogen input [14]. The N_2_O fluxes in cropland ecosystems themselves are inherently complex and heterogeneous due to the combined influence of meteorological elements, soil properties, and cropland management practices [15]. Extreme weather events increase the uncertainty in quantifying N_2_O fluxes from cropland ecosystems. For instance, during periods of extreme precipitation or drought, when the soil water content in cropland is either significantly high or extremely low, N_2_O emissions from the field are reduced, or may even exhibit N_2_O uptake [16]. Therefore, further monitoring and modeling explorations are urgently needed to better understand and quantify how extreme weather events influence N_2_O fluxes in cropland ecosystems. 

With regard to investigation methods, the static chamber-gas chromatograph method is currently the predominant and widely used technique for monitoring N_2_O emissions from cropland at the site scale. The micrometeorological method is suitable for studies at the whole watershed or landscape scales, but its application is limited due to the high cost of instrumentation and the expertise required by operators [17]. Therefore, to quantify the N_2_O emissions from terrestrial ecosystems, several mathematical models have been developed and employed, such as DNDC (denitrification-decomposition), EPIC (environmental policy integrated climate), CERES (crop environment resource synthesis), APSIM (the agricultural production systems simulator), and CLM (community land model) [18,19,20,21,22]. In particular, the CLM model has advantages in simulating crucial processes, such as soil carbon and nitrogen cycles, hydrological processes, and crop growth across various spatial scales [23]. It serves as an effective tool for analyzing and evaluating nitrogen sequestration, as well as nitrogen emissions in terrestrial ecosystems, under current and future scenarios. Previous studies have successfully utilized the CLM model to investigate the effects of irrigation, fertilization, and enhanced rock weathering on N_2_O emissions from cropland ecosystems [24,25,26]. However, it remains uncertain whether, and how, this model performs in exploring the impacts of extreme weather events on N_2_O emissions from cropland ecosystems.

In this study, we monitored environmental factors and measured N_2_O fluxes in a typical cropland ecosystem in the middle and lower reaches of the Yangtze River (MLRYR) in China. During the field observation period from 2020 to 2023, we documented multiple extreme precipitation, drought, and heat events. Then, we constructed and validated a process-based land surface model (CLM5) using the data collected from the field observations. We aimed (a) to analyze the dynamic characteristics of N_2_O fluxes in rice and winter wheat rotation cropland, (b) to investigate the influence of different extreme weather events on N_2_O fluxes in cropland ecosystems and the underlying mechanisms, and (c) to quantify the impacts of the extreme weather events on N_2_O fluxes in cropland ecosystems in the MLRYR under current and future climate scenarios.

## 2. Materials and Methods

### 2.1. Study Sites and Data

**Figure 1:**
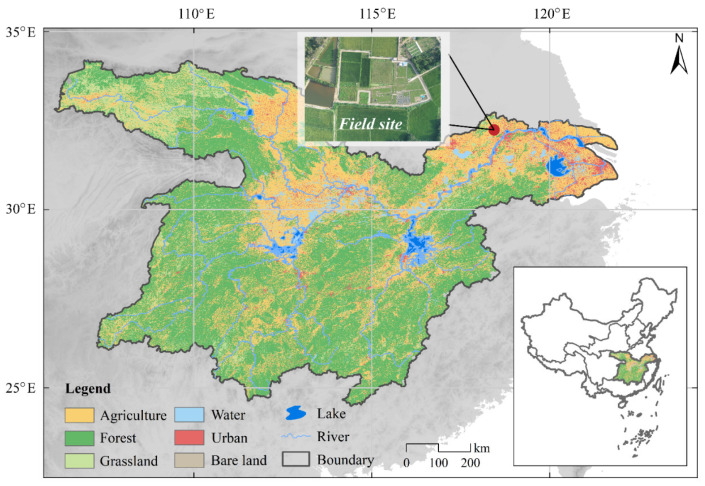
Field location of the study site at the cropland of Chuzhou Scientific Hydrology Laboratory in the middle and lower reaches of the Yangtze River.

**Figure 2:**
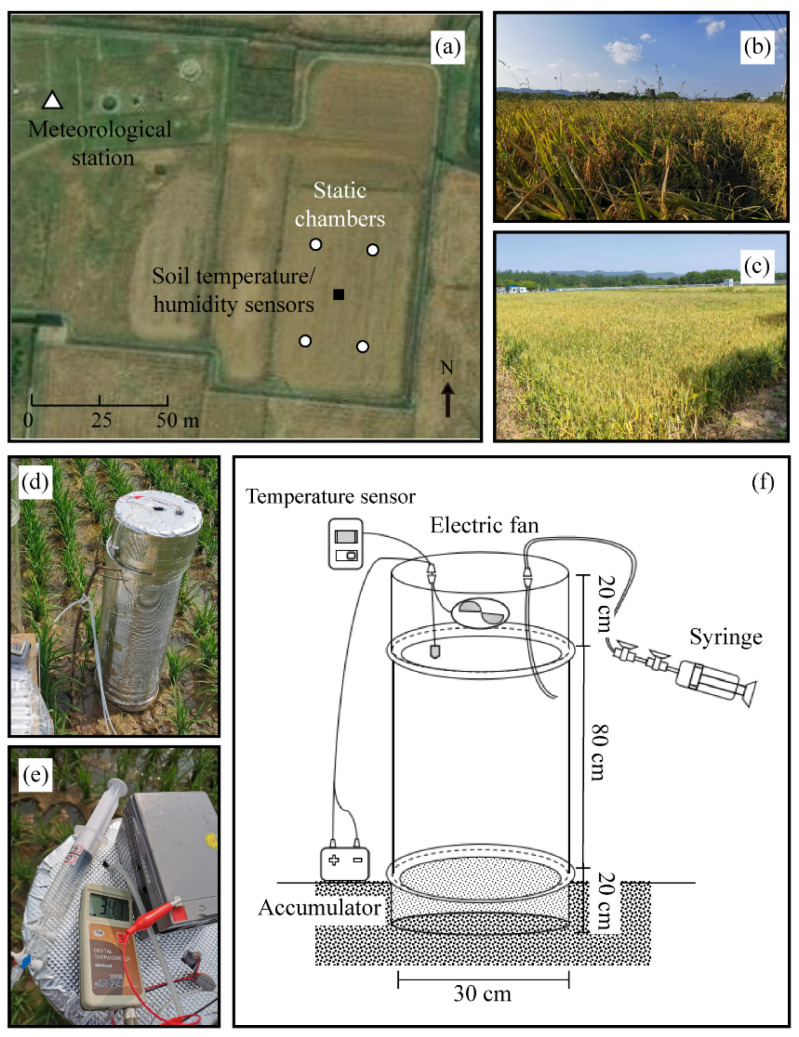
Overview of the study site for the N_2_O fluxes in the cropland ecosystems. (**a**) distribution of sampling points, meteorological station, and water-carbon flux monitoring devices within the field, (**b**,**c**) show the growing seasons of rice and winter wheat at the study site, respectively, and (**d**,**e**) image and (**f**) diagram of the field N_2_O fluxes sampling device. The small white circles, white triangles, and black squares show the location of the static chambers, meteorological stations, and soil temperature and humidity sensors, respectively.

This study was conducted from 1 May 2020 to 5 June 2023, at the experimental fields (32°17′ N, 118°13′ E) of the Chuzhou Scientific Hydrology Laboratory, located in the Anhui province, eastern China, which is part of the MLRYR (Figure 1). The selected site exhibits typical regional characteristics of the MLRYR, which follows a rice-winter wheat rotation cropping system (Figure 2b,c). The soil at this site is silt loam, with a topsoil containing 11.2 g·kg^−1^ organic C, 1.2 g·kg^−1^ total N, 0.4 g·kg^−1^ available P, 17.7 g·kg^−1^ available K, and a mean pH of 6.6. The site has an altitude of 39 m above sea level. It exhibits a typical subtropical monsoon climate with a mean temperature of 15.9 °C, an annual frost-free period of 210 days, and a mean annual precipitation of 1008 mm (1950–2020). The precipitation distribution shows that approximately 70% of the precipitation occurs during the hot-humid season (June–October), while the remaining 30% occurs during the cold-dry season (November–May).

The rice plant (Longliangyou1988) was planted with a row spacing of 35 cm and a nominal density of 14.3 plants·m^−1^ on 17 May 2020, 7 June 2021, and 15 June 2022. The wheat (Zhenmai 9) was sown on 31 October 2020, 20 November 2021, and 31 October 2022, after the rice harvest, at a density of 30 g·m^−1^. During the rice growing period, a total of 167 kg N ha^−1^ (94 kg before rice transplanting and 73 kg at the rice tillering stage) were applied as fertilizer from 2020 to 2023. The fertilizers used were the N-P-K compound fertilizer and urea, respectively. As for the winter wheat growing period, a total of 183 kg N ha^−1^ (79 kg after wheat sowing and 104 kg at the wheat re-greening stage) were applied as fertilizer. Again, the N-P-K compound fertilizer and urea were used as fertilizers, respectively. Irrigation was carried out using an automatic pumping system that sourced water from an adjacent pond. Irrigation was practiced only during the rice growing season and not during the wheat growing season.

To measure the dynamic characteristics of N_2_O fluxes in rice-wheat rotation cropland, static chamber and gas chromatography techniques were employed (Figure 2a) [27]. The opaque chamber was constructed using acrylic materials and was covered with a reflective material (polyester aluminized film and expandable polyethylene) on the outside surface (Figure 2d). The chamber consisted of three parts, a base (30 cm diameter × 20 cm height), a mid-section chamber (30 cm diameter × 80 cm height), and a top cover (30 cm diameter × 20 cm height). The upper portion of both the base and mid-section chambers had 2 × 3 cm cut-out water sinks, which were filled with water to ensure an airtight seal during gas sampling. The base was inserted into the soil before crop planting and it remained fixed throughout the entire observation period. The other two parts of the chamber were used only during the measurements and were removed afterward. Electric fans (F82Y, Pccooler, Shenzhen, China), temperature sensors (JM624, Jinming, Tianjin, China), accumulators, silicone hoses, and 3-way valves were incorporated into the chamber. The specific structures of static chambers are illustrated in Figure 2e,f. In the experimental field, a total of eight static chambers were installed in four groups. Each group consisted of a chamber with plants and a chamber without plants, which were used to measure the N_2_O fluxes in cropland ecosystems and soils, respectively. Gas sampling was conducted between 09:00 and 11:00, which was considered representative of the daily mean of N_2_O fluxes [28]. Gas samples were taken from each chamber headspace using a 60-mL syringe at 0, 15, 30, and 45 min after chamber closure, respectively. The samples were transported back to the laboratory for N_2_O concentration analysis using a gas chromatograph within 24 h (Agilent 8890A, Franklin, CA, USA) [29]. The gas concentration was analyzed using an electron capture detector inside the gas chromatograph. N_2_O fluxes were calculated based on the rate of change in gas concentration, estimated as the slope of a linear regression between time and gas concentration. The results of linear regression with a coefficient of determination (*r*) less than 0.85 were removed from this study.

A meteorological station was installed near the study site to measure various parameters. These included photosynthetically active radiation, atmospheric pressure, wind speed, precipitation, air relative humidity, and air temperature. The measurements were taken at a height of 1.5 m in 5 min intervals (Figure 2a). In addition, volumetric soil water content and soil temperature were continuously monitored at the center of the cropland. Sensors (5TM, METER, WA, USA) were used to collect these data at depths of 5, 10, and 20 cm in 10 min intervals. The meteorological data used to drive the site-scale model included the aforementioned parameters. It also included down-welling shortwave and longwave radiation, which were monitored by the meteorological station. For the regional-scale model, meteorological data, land cover data, and spatial domain data were obtained from the Climatic Research Unit-National Centers for Environmental Prediction (CRUNCEP) datasets (https://svn-ccsm-inputdata.cgd.ucar.edu/trunk/inputdata/atm/, accessed on 16 October 2021). The air-forcing data for the future scenario simulations were obtained from the Coupled Model Intercomparison Project Phase 6 (CMIP6) dataset (https://esgf-node.llnl.gov/search/cmip6/, accessed on 18 October 2021). All simulations were conducted at 30 min intervals, and the regional-scale simulations were performed at a spatial resolution of 0.9° × 1.25°.

### 2.2. Community Land Model

We utilized the community land model version 5 (CLM 5) to simulate the nitrogen cycling process in cropland ecosystems and to reproduce the seasonal variations of N_2_O fluxes within a rotation of rice and winter wheat cropland. CLM 5 is integrated within the community earth system model version 2 (CESM2) and is classified as one of the global gridded crop models (GGCMs) participating in the agricultural model intercomparison and improvement project (AgMIP) [30,31,32]. The model incorporated the CENTURY-based soil carbon (C) pool kinetics to simulate biogeophysical and biogeochemical processes, including plant growth, water cycling, and C and N cycles in natural ecosystems [33,34,35]. CLM5 employs a subgrid hierarchy with multiple land units, columns, and patches embedded within each grid cell to account for variations in biogeophysical and biogeochemical processes and spatial heterogeneity between different land types within the same grid cell [23,36,37]. To better represent cropland ecosystems, crop growth modules based on the AgroIBIS model have been integrated into CLM5. These modules encompass a wide range of crops (such as rice and wheat) and account for multiple processes, as well as various anthropogenic cropland management activities like fertilization and irrigation [38,39].

CLM5 considers soil mineral nitrogen supplied to crops from atmospheric deposition, biological fixation, and fertilization management [26,40]. The model simulates crop phenology process based on daily temperature accumulation, which is divided into three stages: planting to leaf emergence, leaf emergence to grain filling, and grain filling to maturity [31,41]. Throughout the growth process, crops receive nutrients from the soil mineral N pool. Soil mineral nitrogen is lost due to nitrification, denitrification, leaching, and fire events [42]. The soil mineral N in the model consists of NH_4_^+^ and NO_3_^−^, with nitrification from the NH_4_^+^ pool being an input to the NO_3_^−^ pool. Nitrification and denitrification rates are comparable, whereas the N_2_O produced during denitrification far exceeds that produced during nitrification [43]. Most of the N_2_O emissions from cropland ecosystems originate from denitrification. The overall N stocks in these ecosystems depend on the dynamic balance between N cycling within plants and N cycling within the soil N pool.

Models that incorporate biogeophysical and biogeochemical components require a long-term spin-up period for their C and N pools to reach equilibrium [39,44]. To ensure a stable exchange of matter and energy in the initial simulation conditions, we conducted an accelerated decomposition spin-up run for 1000 years, followed by a normal spin-up run for 500 years before initiating the site simulation [34,40,45]. For the site-scale simulation, model parameters were calibrated to match the simulated N_2_O fluxes from the cropland with observations. In this study, the parameterization involved eight parameters, primarily related to three modules: crop phenology, field management, and N fixation and uptake. The first two modules consisted of six parameters, such as the minimum planting time and temperature, maximum number of planting days, maximum leaf area index, irrigation duration, and amount of fertilizer applied. The calibration process and specific parameter settings for these parameters are detailed in Table S1 and described in Xia et al. (2021) [46]. The parameters related to the N fixation and uptake module are *Nitroph* and *Denitrat*, representing cropland soil pH and the ratio of N_2_ to N_2_O in the denitrification process, respectively. Specifically, the value of the parameter *Nitroph* was adjusted to 6.6 based on measured soil pH in the field. Then, the parameters *Denitrat* were adjusted based on observed N_2_O fluxes from the cropland. Following the parameter adjustment, simulated N_2_O from denitrification during the rice growing season decreased, while simulated N_2_O from denitrification during the wheat growing season increased.

## 3. Results and Discussion

### 3.1. Meteorological and Soil Variables

For the observation period 2020–2023, the mean annual air temperature was 15.6 °C, with the monthly temperature ranging from 1.9 °C (December 2022) to 28.7 °C (July 2022, Figure 3). The mean daily air temperature for the rice and winter wheat growing season was 25.2 °C and 10.1 °C, respectively. The site received annual precipitation of 1087.4 mm during the study period, with 67% occurring between June and October during the rice growing season. The largest monthly precipitation was observed in July 2020, totaling 411.6 mm, while the smallest monthly precipitation was observed in December 2021, totaling 2.5 mm. The rice (winter wheat) growing season in 2022 (2020–2021) was wetter with a daily average relative humidity of 83% (75%), and in 2020 (2022–2023) was drier with an average daily relative humidity of 81% (74%). In addition, the daily average photosynthetically active radiation during the growing season of rice and winter wheat was 368.1 µmol·s^−1^·m^−2^ and 261.2 µmol·s^−1^·m^−2^, respectively.

**Figure 3:**
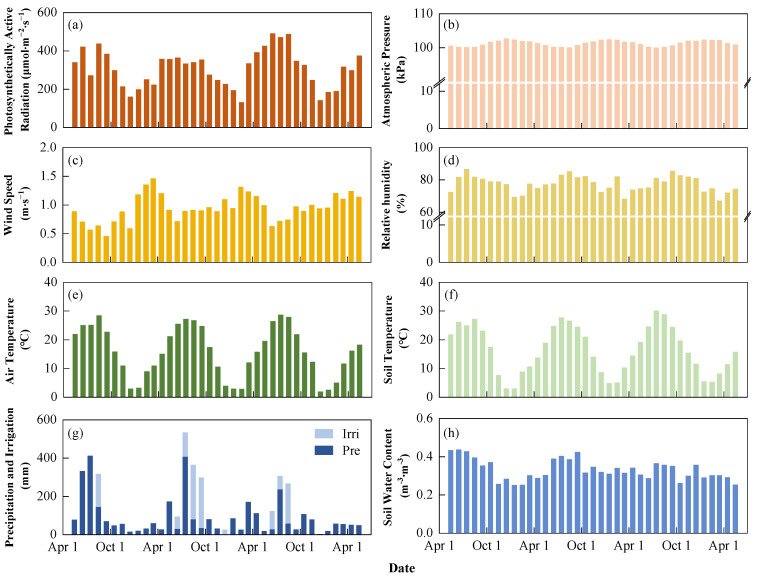
Monthly meteorological and soil temperature and moisture data from this study site. (**a**–**e**) show monthly average photosynthetically active radiation, atmospheric pressure, wind speed, relative humidity, and air temperature, respectively. (**f**,**h**) indicate the average soil temperature and soil water content of the cultivated layer, respectively. (**g**) denotes monthly cumulative precipitation and irrigation. “Irri” and “Pre” represent irrigation and precipitation, respectively.

Daily average soil temperature in the cultivated layer (depth of 0–20 cm) followed the same pattern as air temperature, with greater differences between the two from November to January. In winter, the mean daily soil temperature ranged from −1.2 °C to 17.1 °C, while the mean daily air temperature ranged from −6.1 °C to 20.3 °C (Figure 3e,f). The mean daily soil water content (SWC) measured in the cultivated layer for the irrigated rice growing season was 0.38 m^3^·m^−3^, while the corresponding SWC for rain-fed winter wheat was 0.30 m^3^·m^−3^. Specifically, SWC increased sharply after irrigation management during the pre-transplanting stage of rice and remained high throughout the summer, then gradually decreased after the rice harvest. From April to May, which corresponded to the heading to maturing stage of rain-fed wheat, the SWC remained relatively low at 0.28 m^3^·m^−3^. This can be attributed to the limited precipitation and the higher water requirements of wheat during this period.

### 3.2. Observed and Modeled N_2_O Fluxes in Cropland

**Figure 4:**
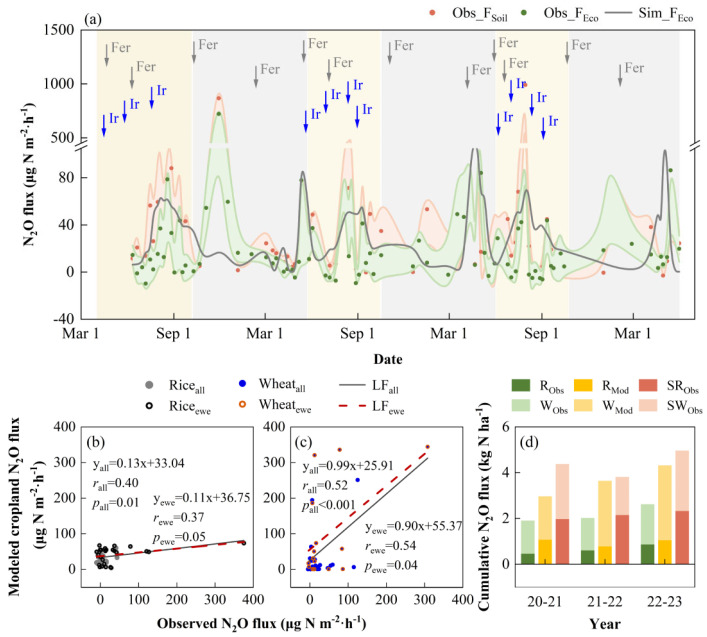
Observed and modeled nitrous oxide (N_2_O) fluxes at the study site. (**a**) Observed and modeled N_2_O fluxes in 2020–2023. “Fe” and “Ir” represent fertilization and irrigation activities scheduled during the rice and wheat growing season. “F_Soil_” and “F_Eco_” represent seasonal changes in N_2_O fluxes in cropland without and with crops, respectively. The green−color−filled and red−color−filled areas show the corresponding standard errors. Correlations between the observed and modeled N_2_O fluxes during rice (**b**) and winter wheat (**c**) growing season at the study site. “LF_all_” and “LF_ewe_” represent linear fits between observed and modeled N_2_O fluxes for the entire growing season and the period of extreme weather events, respectively. (**d**) Cumulative N_2_O fluxes in rice and wheat fields from 2020 to 2023. “R_Obs_” and “W_Obs_” represent the observed N_2_O fluxes during the growing seasons of rice and wheat, respectively, while “R_Mod_” and “W_Mod_” represent the corresponding modeled values. “SR_Obs_” and “SW_Obs_” represent the observed N_2_O fluxes of bare soil during the growing seasons of rice and wheat, respectively.

The N_2_O fluxes in the cropland ecosystem of rice and winter wheat rotation exhibited a clear seasonal pattern (Figure 4a). Very weak N sinks were observed during the rice jointing-booting and grain filling stages, with an average N_2_O flux of −3.4 µg N m^−2^·h^−1^, while N sources were evident during the rice tillering, booting-grain filling, and maturing stages, with an average N_2_O flux of 24.9 µg N m^−2^·h^−1^. Throughout the winter wheat growing seasons, the cropland ecosystems primarily emitted N_2_O to the atmosphere, with an average N_2_O flux of 29.4 µg N m^−2^·h^−1^. Peak N_2_O fluxes in cropland ecosystems typically occur after the application of basal and follow-up fertilizers. For instance, we observed peak N_2_O emissions (722.0 µg N m^−2^·h^−1^) from the cropland ecosystem after the application of basal fertilizer on 31 October 2020, before wheat sowing. Similarly, after the application of rice tiller fertilizer on 22 June 2022, the cropland experienced very high N_2_O emissions (375.4 µg N m^−2^·h^−1^). Chen et al., (2019) also found that peak N_2_O emissions were generally captured after the application of basal, tillering, and jointing fertilizers in field experiments on rice-barley rotation cropland in the MLRYR in China, which is consistent with our observations [47]. In addition to cropland ecosystems, we measured the dynamics of N_2_O fluxes from bare cropland soils. Overall, bare soil acted as a source of N, with N_2_O emissions increasing with temperature. Unusually high emission peaks often occur after fertilizer management. During the rice and wheat growing seasons, the average soil N_2_O emissions were 43.3 and 44.8 µg N m^−2^·h^−1^, respectively.

In general, the cropland ecosystem during the rice growing season exhibited a weak N source, with an average annual cumulative net N_2_O fluxes of 64.6 mg N m^−2^. The cropland showed a relatively greater intensity of N source during the wheat growing season, with an average annual cumulative net N_2_O fluxes of 153.4 mg N m^−2^. Several meta-analyses have shown that N fluxes during the growing season in irrigated rice fields in China and other Asian regions varied between −63.6 and 56.9 µg N m^−2^·h^−1^, which depended on factors such as soil type, soil chemical characteristics, soil moisture status, and N fertilizer management [48,49]. In this study site, we found that the average N_2_O fluxes in cropland ecosystems during the rice growing season ranged from 15.9 to 34.4 µg N m^−2^·h^−1^, which falls within the aforementioned range of N_2_O fluxes. In a field experiment conducted in rice-wheat rotation cropland in the MLRYR in China, the cumulative N_2_O emissions from cropland ecosystems during the wheat growing season were reported as 388 ± 65 mg N m^−2^ [50], which is higher than our observations (153 ± 115 mg N m^−2^). This difference can be attributed to variations in N fertilizer application rates between the two sites. The experiment by Liu et al. (2010) implemented an application rate of 250 kg N ha^−1^, while our study used a corresponding rate of 183 kg N ha^−1^. Furthermore, the cumulative net N_2_O fluxes from rice-wheat rotation cropland were 1.9, 2.0, and 2.6 kg N ha^−1^ in the years 2020–2021, 2021–2022, and 2022–2023, respectively (Figure 4d). In contrast, the average annual N_2_O emissions from cropland soils during the observation period were larger, approximately 0.9–1.3 times higher than the annual N_2_O emissions from cropland ecosystems.

The model successfully captured the seasonal dynamics of the observed N_2_O fluxes from cropland ecosystems (Figure 4a). The correlation coefficient (*r*) between the observed and simulated N_2_O fluxes was 0.40 (*p* = 0.01) and 0.52 (*p* < 0.01) during the growing seasons of rice and wheat, respectively (Figure 4b,c). Although we observed occasional high peaks of N_2_O emissions during the crop growing season as a result of fertilization and irrigation practices, there were not accurately captured by the CLM5 model. Nevertheless, the model generally replicated the seasonal trends observed in N_2_O fluxes at this study site. In addition, the model’s simulated annual cumulative N_2_O emissions from cropland ecosystems were approximately 3.64 kg N ha^−1^, around 0.4–0.9 times higher than the observed values. This discrepancy may be attributed to the temporal discontinuity in the field measurements of N_2_O fluxes, which were collected using static chamber-gas chromatograph analysis, while the model simulates N_2_O fluxes at hourly intervals. Therefore, the simulated annual cumulative N_2_O fluxes tend to be higher than the observed values at our study site. The correlation coefficient (*r*) between the observed and simulated N_2_O fluxes during extreme weather events was 0.37 (*p* = 0.01) and 0.54 (*p* = 0.04) during the growing seasons of rice and wheat, respectively. This suggests that the model can generally simulate the impacts of extreme weather events on N_2_O fluxes in cropland ecosystems.

### 3.3. Impact of Extreme Weather Events on N_2_O Emissions

In this study, we defined an extreme heat event as a period of more than three consecutive days during the crop growing season where the daily maximum air temperature exceeded 35 °C. Our monitoring results revealed three such extreme heat events: 15–19 August 2020, 10–15 July 2022, and 11–15 August 2022. The average daily maximum air temperatures recorded during these events were 36.5, 35.7, and 36.7 °C, respectively. Notably, all of these events occurred during the rice growing season. Similarly, we identified an extreme precipitation event as a single day with cumulative precipitation exceeding 50 mm, along with a monthly precipitation level that more than doubled compared to the multi-year average. An extreme drought event was identified as a single month with precipitation less than one-quarter of the multi-year average. Based on these definitions, we observed two extreme precipitation events in June 2020 during the rice growing season and March 2022 during the wheat growing season. The monthly precipitation during these events was 1.4 and 1.2 times higher than the multi-year average, respectively. We also documented three extreme drought events during the June 2021 and June 2022 rice growing seasons, as well as the May 2022 wheat growing season. The monthly precipitation during these events corresponded to only 22%, 21%, and 21% of the multi-year average, respectively.

#### 3.3.1. Extreme Heat Events

**Figure 5:**
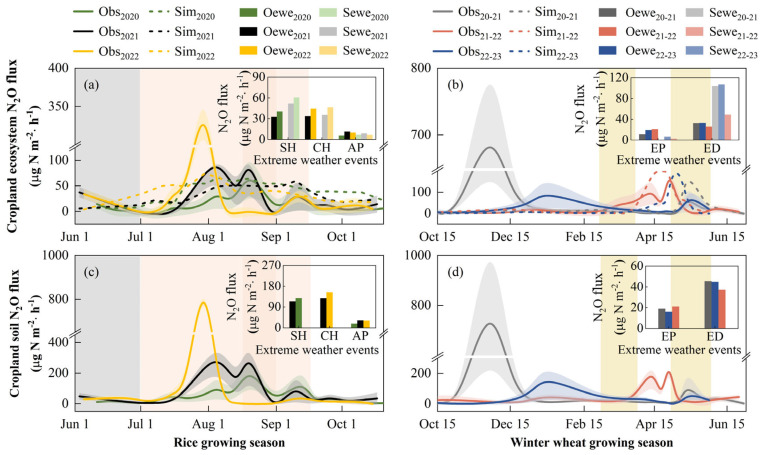
Influences of extreme weather events on cropland ecosystem nitrous oxide (N_2_O) fluxes and cropland soil N_2_O fluxes at the study site. Observed (solid lines) and simulated (dash lines) cropland ecosystem and soil N_2_O fluxes dynamics in the rice (**a**,**c**) and winter wheat (**b**,**d**) rotation cropland for three observation years. The color−filled areas show the corresponding standard errors of the measurements. “SH”, “CH”, and “AP” represent a single extreme heat event, two consecutive extreme heat events, and abnormal precipitation (extreme precipitation and drought) events during the rice growing season, respectively. “EP” and “ED” represent extreme precipitation and drought events during the wheat growing season, respectively. “Oewe” and “Sewe” represent the observed and simulated average N_2_O fluxes during periods of extreme weather events, respectively. The gray and orange rectangular areas represent abnormal precipitation events and extreme heat events that occurred during the rice growing season, respectively. The yellow rectangular areas represent abnormal precipitation events that occurred during the wheat growing season.

In general, extreme heat events led to an increase in N_2_O emissions from rice fields (Figure 5). An extreme heat event lasting 5 days in August 2020, during the rice heading-filling period, resulted in observed average N_2_O fluxes of 40.4 µg N m^−2^·h^−1^ in cropland ecosystems, representing a 23% rise compared to the same period in 2021 (32.8 µg N m^−2^·h^−1^). Similarly, two consecutive extreme heat events transpired during the early stages of rice reproductive growth in July and August 2022, lasting 6 and 5 days, respectively. During this period, the observed average N_2_O fluxes in cropland ecosystems reached 44.6 µg N m^−2^·h^−1^, indicating a 32% increase compared to the same period in 2021 (33.9 µg N m^−2^·h^−1^). The model also captured the impact of extreme heat events on N_2_O fluxes in cropland ecosystems. Specifically, extreme heat events in August 2020 and July–August 2022 resulted in a 17% and 31% increase in modeled N_2_O emissions from rice fields, respectively, compared to the same period in 2021. Furthermore, we observed that extreme heat events affected bare soil in rice fields. Specifically, the extreme heat event in August 2020 and the two consecutive events in July–August 2022 resulted in average bare soil N_2_O emissions that were 13% and 20% higher than the same period in 2021, respectively. A controlled experiment also demonstrated that the occurrence of two and three extreme heat events significantly increased cumulative N_2_O emissions from bare soils by 1.5 and 4.1 times, respectively, compared to the controls conditions [51]. These findings align with our observations. Heat events can potentially enhance N_2_O emissions by raising soil temperatures and stimulating soil nitrification and denitrification processes [52,53]. 

Notably, heat events not only raise soil temperatures, which directly affect N_2_O emissions from cropland soils, but may also influence plant behavior in terms of nitrogen oxides uptake. Specifically, heat stress can negatively impact physiological functions in plant roots, including the formation and activity of root hairs; it can also lead to increased secretion of solutes such as organic acids by the roots, altering the osmotic pressure within the root system [52,54,55,56]. As a result, heat events promote nitrification and denitrification in the field, while also weakening the N uptake capacity of the rice root system, which ultimately leads to an increase in N_2_O emissions from the cropland ecosystem.

#### 3.3.2. Extreme Precipitation and Drought Events

Extreme precipitation events have shown contrasting effects on N_2_O fluxes in different crop fields, which reduced N_2_O emissions during the rice growing season and increased N_2_O emissions during the wheat growing season. During the rice growing season, we observed an extreme precipitation event in June 2020, while extreme drought events occurred in June 2021 and June 2022. In June 2020, the observed average N_2_O fluxes in rice fields were 5.8 µg N m^−2^·h^−1^, which was 49% and 42% lower compared to the corresponding N_2_O fluxes in June 2021 (11.4 µg N m^−2^·h^−1^) and June 2022 (10.0 µg N m^−2^·h^−1^), respectively. Similarly, bare soil N_2_O emissions in June 2020 decreased by 45% and 44% compared to the same period in 2021 and 2022, respectively. In rice fields, excessively high SWC negatively impacts N_2_O diffusion [57]. Additionally, it restricts the growth and metabolic activities of microorganisms in the soil, thereby impeding N_2_O production and transformation [9,58]. In contrast, extreme precipitation events during the wheat growing season elevated the observed average N_2_O fluxes from cropland ecosystems by 36% compared to the corresponding multi-year average. In rain-fed wheat cropland, short-term and high-intensity precipitation rapidly increases SWC, promoting organic matter decomposition and stimulating nitrification and denitrification processes, while facilitating the release of N_2_O from the soil to the atmosphere [59,60,61]. The simulation results indicated that the extreme precipitation event in June 2020 led to a 22% reduction in N_2_O emissions from rice fields compared to the same period in 2021, which aligns with our observations. However, there were no significant differences in the simulated average N_2_O fluxes from rice fields between June 2020 (6.9 µg N m^−2^·h^−1^) and 2022 (6.8 µg N m^−2^·h^−1^). Similarly, during the wheat growing season, the extreme precipitation event in March 2022 resulted in only a 2% increase in average N_2_O fluxes compared to the multi-year average for that month. Therefore, although the CLM5 model effectively reproduced the seasonal variation of N_2_O fluxes in cropland ecosystems, additional validation is necessary when applying the model to investigate the impacts of extreme precipitation events on N_2_O fluxes in cropland. Plants exhibit the potential to adapt to extreme weather conditions, while the current model employs a relatively simple structure of crop physiological modules. The description of plant physiological characteristics, particularly plant water dynamics, is too simple in the model. Incorporating relevant mechanisms and processes may improve the model’s ability to simulate the impacts of extreme precipitation events on cropland N_2_O fluxes.

An extreme drought event occurred during the wheat growing season in May 2022. The average N_2_O emissions for that month was 26.1 µg N m^−2^·h^−1^ in cropland ecosystems, a 20% decrease in N_2_O emissions compared to the multi-year average (32.7 µg N m^−2^·h^−1^), with a corresponding 18% reduction in N_2_O emissions from bare soil. The findings of Gelfand et al., (2015), based on a combination of field observation experiments and indoor control experiments, support our observations. They demonstrated that drought conditions significantly decreased N_2_O emissions from cropland [62]. Our model also captured the impact of extreme drought events on N_2_O emissions from wheat fields. In May 2022, the simulated average N_2_O emissions were reduced by 53% compared to the multi-year average. Thus, extreme precipitation and drought events increased and decreased N_2_O emissions from rain-fed wheat fields, respectively, while extreme precipitation events led to a decrease in N_2_O emissions from irrigated rice fields.

### 3.4. Impact of Extreme Weather Events on N_2_O Emissions in the MLRYR

**Figure 6:**
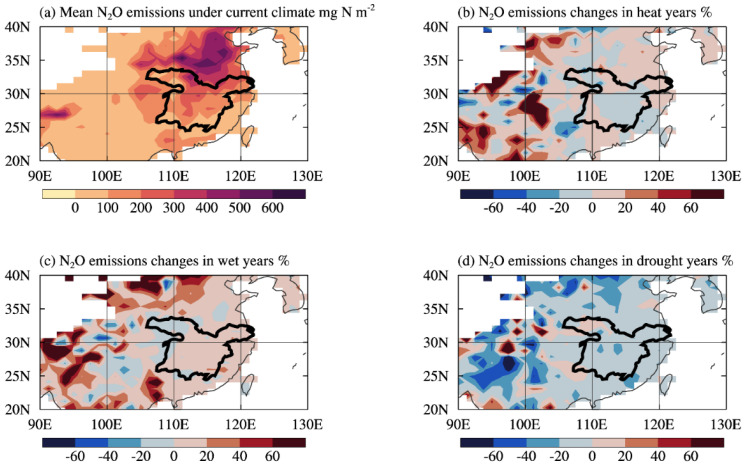
Spatial distribution of average annual N_2_O emissions (**a**) and corresponding changes (**b**–**d**) from rice and wheat cropland ecosystems in the Middle and Lower Reaches of the Yangtze River from 1997 to 2016 under the current climate scenario.

N_2_O emissions from rice and wheat cropland in the MLRYR exhibited significant spatial heterogeneity (Figure 6a). The average annual cumulative N_2_O emissions in the region ranged from 16.5 to 636.7 mg N m^−2^ between 1997 and 2016, with an average of 207.8 mg N m^−2^. The highest N_2_O emissions were observed in the northern part of the MLRYR, mainly due to the higher concentration of agricultural land near the mainstream of the Yangtze River. N_2_O emissions from cropland in the MLRYR showed a decreasing trend from north to south. In our regional simulation, the annual cumulative N_2_O emissions from croplands within the grid (0.9° × 1.25°) that corresponds to our study site were estimated at 237.5 mg N m^−2^, which showed a 9% absolute difference compared to the observed value of 218.0 mg N m^−2^. After calibrating the model parameters, the simulated N_2_O emissions from cropland in the MLRYR matched well with the observations. Under future climate scenarios, the model predicts a significant increase in N_2_O emissions from cropland in the MLRYR (Figure S1a). Between 2081 and 2100, the annual cumulative N_2_O emissions in the region are projected to range from 26.5 to 715.0 mg N m^−2^, with an average of 303.4 mg N m^−2^. Particularly in the northern part of the MLRYR, where croplands are prevalent, the future annual cumulative N_2_O emissions are estimated to increase by 42% compared to the current climate scenario.

In the regional simulations, the hottest two years during the crop growing season (10% of the 20 years) were defined as heat years for both current and future climate scenarios. Similarly, the two years with the highest and lowest precipitation during the crop growing season were defined as wet and drought years, respectively. Under the current climate scenario, N_2_O emissions from cropland ecosystems in the MLRYR generally increased in wet years compared to multi-year averages, while they decreased in drought years, consistent with our site observations (Figure 5 and Figure 6c,d). During heat years, annual cumulative N_2_O emissions of the grid where our study site were located in the regional simulation increased by 8% compared to the multi-year average, which aligned with site observations (Figure 6b). However, in the central part of the MLRYR where agricultural land is less prevalent, N_2_O emissions decreased by approximately 3% from the multi-year average during heat years. This pattern may be attributed to elevated temperatures intensifying evapotranspiration from the cropland ecosystem, leading to reduced SWC. Decreased SWC negatively affected soil oxidation and reduction under low oxygen conditions, thereby reducing N_2_O production [63]. Notably, the simulation under the future climate scenario predicts a substantial 17% increase in annual cumulative N_2_O emissions from the primary rice- and wheat-growing regions in the MLRYR during heat years, significantly higher than the increase under the current climate scenario (2%, Figure S1b). Considering that extreme heat events will primarily impact cropland ecosystems in the MLRYR in the future [12], it is crucial to enhance monitoring and modeling efforts to further investigate the effects of extreme heat events on N_2_O emissions from cropland ecosystems in future research.

## 4. Conclusions

In this study, we measured N_2_O fluxes in typical rice-winter wheat rotation cropland in the MLRYR in China from 2020 to 2023. Various extreme weather events, including extreme precipitation, drought, and heat, were recorded during the observation period. Then, we combined field observations with the process-based CLM5 model to investigate the dynamic characteristics of N_2_O fluxes in cropland ecosystems and their responses to extreme weather events. The key findings include:

(a) The cropland with rice and winter wheat rotation exhibited a source of N_2_O, with annual cumulative N_2_O emissions ranging from 190.4 to 261.8 mg N m^−2^. N_2_O emissions from rice cropland peaked during the rice booting-filling stage, contributing 29% to the annual cumulative emissions. N_2_O emissions from wheat cropland reached the peak during the maturing stage, accounting for 71% of the annual cumulative emissions.
(b) There was a 23% to 32% increase in observed N_2_O emissions from cropland ecosystems during extreme heat events which likely resulted from enhanced nitrification and denitrification processes and weakened nitrogen uptake capacity of the rice root system. N_2_O emissions from irrigated rice cropland during extreme precipitation events were 45% lower compared to those during extreme drought events. In the growing seasons of rain-fed wheat, extreme precipitation events increased observed N_2_O emissions by 36%, while extreme drought events decreased N_2_O emissions by 20%.
(c) The simulations reproduced the seasonal dynamics of N_2_O fluxes from cropland ecosystems well. Regional simulations demonstrated that the annual cumulative N_2_O emissions from rice and wheat cropland in the MLRYR were 207.8 mg N m^−2^, and N_2_O emissions are projected to increase in the future (303.4 mg N m^−2^). Furthermore, future extreme heat events are expected to enhance the N_2_O emissions. Considering the complexity and uncertainty in the responses of N_2_O emissions from cropland ecosystems to extreme weather events, exceptional caution should be taken when utilizing the model to investigate the impacts of extreme events on N_2_O emissions from cropland ecosystems.

To accurately assess the complex impacts of extreme weather events on N_2_O fluxes in cropland ecosystems, long-term field measurements and studies combining observations and simulations are required.

## Data Availability

The present regional meteorological forcing, domain, and land coverage data were obtained from the CRUNCEP data sets (https://svn-ccsm-inputdata.cgd.ucar.edu/trunk/inputdata/atm/, accessed on 16 October 2021). The future model-driven data were derived from the Coupled Model Intercomparison Project Phase 6 (CMIP6) dataset (https://esgf-node.llnl.gov/search/cmip6/, accessed on 18 October 2021). The site meteorological data and N_2_O flux data of the cropland will be made available upon request.

## Supplementary Materials

The following supporting information can be downloaded at: https://www.mdpi.com/article/10.3390/plants13010025/s1, Figure S1: Spatial distribution of average annual N_2_O emissions and corresponding changes from rice and wheat cropland ecosystems in the Middle and Lower Reaches of the Yangtze River from 2081 to 2100 under the future climate scenario; Table S1: Key parameters in the crop phenology, field management, and nitrogen fixation and uptake modules used for the modified CLM5 at this study site.
