# Supplementary material for: Effects of Extreme Weather Events on Nitrous Oxide Emissions from Rice-Wheat Rotation Croplands
Source: Plants (Basel). 2023 Dec 20;13(1):25. doi: 10.3390/plants13010025 (PMC10780663; doi:10.3390/plants13010025)
Supplement: Supplementary file 1 [file plants-13-00025-s001.zip › plants-2752358-supplementary.pdf]

Effects of extreme weather events on nitrous oxide emissions from rice-wheat rotation croplands

Ye Xia[1], Congsheng Fu[1,2], Aimin Liao[3], Huawu Wu[1], Haohao Wu[1], Haixia Zhang[1]

[1]Key Laboratory of Watershed Geographic Sciences, Nanjing Institute of Geography and Limnology, Chinese Academy of Sciences, Nanjing 210008, China

[2]Collaborative Innovation Center on Forecast and Evaluation of Meteorological Disasters (CIC-FEMD), Nanjing University of Information Science & Technology, Nanjing 210044, China

[3]Chuzhou Scientific Hydrology Laboratory, Nanjing Hydraulic Research Institute, Chuzhou 239080, China

Contents of this file

Figures S1
Table S1

Introduction

Figure S1 displays spatial distribution of average annual N_2O emissions and corresponding changes from rice and wheat cropland ecosystems in the Middle and Lower Reaches of the Yangtze River from 2081 to 2100 under the future climate scenario. Table S1 shows key parameters in the crop phenology, field management, and nitrogen fixation and uptake modules used for the modified CLM5 at this study site.

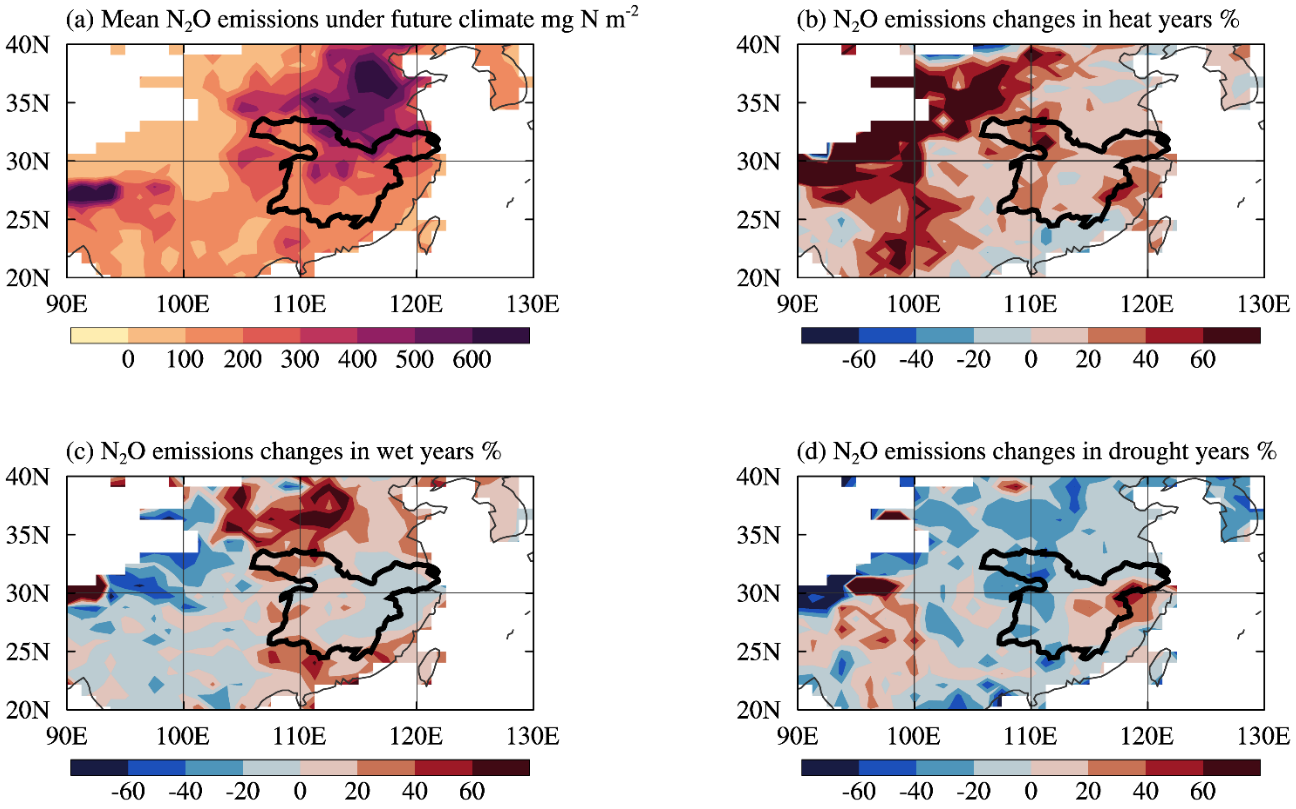

Figure S1. Spatial distribution of average annual N_2O emissions (a) and corresponding changes (b – d) from rice and wheat cropland ecosystems in the Middle and Lower Reaches of the Yangtze River from 2081 to 2100 under the future climate scenario.

Table S1. Key parameters in the crop phenology, field management, and nitrogen fixation and uptake modules used for the modified CLM5 at this study site.

Parameters		Description	Rice	Winter wheat
Crop phenology	*minplantdate* (DOY)	Minimum planting date	60	273
	minptemp (K)	Minimum planting temperature	283.15	279.15
	mxmat (days)	Maximum days for growing	135	320
	laimx ($m^2 \cdot m^{-2}$)	Maximum leaf area index	7	6.5
Field management	*Irriglength* (sec)	Duration of irrigation per day	28800	--
	Fertnitro (g N m^{-2})	Fertilizer application on cropland	16.7	18.3
N fixation and uptake	*Nitroph*	Cropland soil pH value	6.6	6.6
	Denitrat	Ratio coefficient of N_2 to N_2O in denitrification process	10.40	0.95

Note: DOY: day of year.
